# Dynamic Network Biomarker Analysis Reveals the Critical Phase Transition of Fruit Ripening in Grapevine

**DOI:** 10.3390/genes13101851

**Published:** 2022-10-13

**Authors:** Tengfei Wang, Huixiang Peng, Yingying Cao, Jing Xu, Yuhong Xiong, Kangchen Liu, Jing Fang, Fang Liu, Aidi Zhang, Xiujun Zhang

**Affiliations:** 1 Key Laboratory of Plant Germplasm Enhancement and Specialty Agriculture, Wuhan Botanical Garden, Chinese Academy of Sciences, Wuhan 430074, China; 2 Center of Economic Botany, Core Botanical Gardens, Chinese Academy of Sciences, Wuhan 430074, China; 3 University of Chinese Academy of Sciences, Beijing 100049, China

**Keywords:** systems biology, network analysis, dynamic network biomarker, phase transition, fruit development

## Abstract

Grapevine (*Vitis* *vinifera* L.) fruit ripening is a complex biological process involving a phase transition from immature to mature. Understanding the molecular mechanism of fruit ripening is critical for grapevine fruit storage and quality improvement. However, the regulatory mechanism for the critical phase transition of fruit ripening from immature to mature in grapevine remains poorly understood. In this work, to identify the key molecular events controlling the critical phase transition of grapevine fruit ripening, we performed an integrated dynamic network analysis on time-series transcriptomic data of grapevine berry development and ripening. As a result, we identified the third time point as a critical transition point in grapevine fruit ripening, which is consistent with the onset of veraison reported in previous studies. In addition, we detected 68 genes as being key regulators involved in controlling fruit ripening. The GO (Gene Ontology) analysis showed that some of these genes participate in fruit development and seed development. This study provided dynamic network biomarkers for marking the initial transcriptional events that characterizes the transition process of fruit ripening, as well as new insights into fruit development and ripening.

## 1. Introduction

Grapevine (*Vitis vinifera* L.) is an important horticultural crop with huge economic value, and is used for dried raisins, juice, fresh fruit, and vinification [1,2,3]. Grapevine fruit (berry) development is a complex biological event involving a phase transition from pre-ripe to mature. The tipping point of the phase transition separates the whole process into two phases [4,5]. The pre-phase is a pre-ripe phase with an increase in fruit size and the synthesis of organic acids [6]. Afterwards, a short period characterized by the initiation of sugar accumulation is referred to as the veraison stage, i.e., the phase transition from the pre-ripe to mature stage of grapevine fruit development. The post-phase after veraison constitutes an acid-to-sugar process [7,8]. In the phase transition stage, an important phenotype is the rapid coloring of berries in red and black grape varieties. However, this is not obvious in green and white grape varieties. Understanding the timing and order of the molecular events for fruit ripening is critical for grapevine fruit storage and quality improvement. However, the regulatory mechanism for the critical phase transition of fruit ripening from immature to mature in grapevine remains poorly understood [9,10].

In contrast toclimacteric fruits such as tomato and apple [11], non-climacteric fruits such as grape and strawberry do not have a typical respiratory peak and a single switch for regulating ripening [12,13,14]. Some studies have shown that abscisic acid (ABA), brassinosteroids (BRs) and ethylene play important roles in the grapevine fruit ripening process, and several hormones interact to promote grapevine fruit ripening [15,16,17]. Identification of key genes that regulate the grapevine fruit development helps to understand the regulatory mechanism of critical phase transition of berry ripening in non-climacteric fruits [18].

In transcriptome analysis for deciphering complex biological processes, network-based analysis methods have been widely used in plant bioinformatics [19,20,21]. There are also some studies using network analysis to investigate fruit ripening and identify the key genes that regulate fruit ripening [22,23]. Current network inference methods in bioinformatics are accurate enough. For example, information theory-based methods have been developed to infer directed gene regulatory networks, such as PCACMI [24], NARROMI [25], CMI2NI [26], PCAPMI [27], and RSNET [28]. The weighted correlation network analysis (WGCNA) based on Pearson’s correlation coefficient is the most popular network-based method, and is able to select genes and detect modules for some functions [29].

The mechanism of phase transition has been widely studied and proved to be important in some biological sciences. In medical science, the transition in cancer development from tumor to cancer has been studied for pre-disease prevention [30,31]. In plant science, the transition of flower development and the transition of plant growth from growth to defense in *Arabidopsis* have been studied in our previous works [32,33]. In non-model-plant sweet potato, the phase transition from pre-swelling to storage roots has been studied, and the regulator IbNAC083 was discovered to play an important role in the initiation and development of storage root [34]. Abrupt and non-smooth are two obvious traits of the phase transition in biological processes. In the transition state, the regulatory networks fluctuated violently within the system, resulting in the fragility and state change of the system [35].

In this work, to identify the key molecular events controlling the critical phase transition of grapevine fruit ripening, we performed an integrated dynamic network analysis of time-series transcriptomic data of grapevine fruit ripening. On the basis of our results, a DNB (dynamic network biomarker) module with 68 genes was identified for the key regulators controlling the phase transition of fruit ripening in grapevine. In addition, the third time point was detected as the critical transition point of fruit ripening. Using both GO (gene ontology) functional enrichment and literature analysis, these genes were studied and proved to be involved in fruit and seed development. With the dynamic network analysis of fruit ripening, we provided the dynamic network biomarkers to mark the initiation of transcriptional events that characterizes the transition process of fruit ripening as well as a new insight into fruit ripening in grapevine.

## 2. Materials and Methods

### 2.1. Data Collection and Pre-Processing

Berry samples from fruit setting to full maturity of the grapevine were collected from Cabernet Sauvignon grown over three consecutive vintages in the years 2012, 2013 and 2014. The samples for every 7 to 10 days were collected by using a randomized block method. The raw reads of RNA-seq data (PRJNA386889) and gene expression data (GSE98923) are accessible in the database of NCBI websites http://www.ncbi.nlm.nih.gov/sra/(accessed on 10 August 2020) and http://www.ncbi.nlm.nih.gov/geo/(accessed on 21 August 2020.

To assess the quality of raw reads, the FastQC(v0.11.6) tool was first used for quality control. With the reference genome of *Vitis vinifera* L., the reads were aligned using the mapping tool HISAT (V2.1.0) [36]. The Reads Per Kilo base of exon model per Million mapped reads (RPKM) values for all genes in each sample were quantified using StringTie (V1.3.3b). To obtain unbiased gene expression levels, we counted the median values of four technical replicate samples. Only the genes with effective abundance, i.e., RPKM>1, were used for further analysis.

### 2.2. Differential Gene Expression Analysis

To determine the differentially expressed genes (DEGs), we implemented an empirical Bayesian hierarchical model-based R package—EBSeq (V0.36)—on gene expression data [37]. With the results, two parameters fold change (FC) >2 and false discovery rate (FDR) <0.05, were used to identify significant DEGs.

### 2.3. Functional Enrichment Analysis and Visualization

To assess the function of the grape genes, we used the protein evolutionary relationships-based tool PANTHER (http://pantherdb.org) for gene annotation and functional classification [38]. The significant GO terms were selected with a *p*-value threshold of 0.05. The R package ggplot2 (V3.3.5) was used to visualize the results of the significant enrichment analysis.

### 2.4. Network Analysis and Visualization

To investigate the function of the identified genes, we constructed a gene network of these genes and their surrounding genes with the background network from the STRING database. To filter out the network, the threshold of the comprehensive score parameter was set as 0.6 [39]. The PPI (protein–protein interactions) network was included as known and predicted interactions between the protein-coding genes with a confidence score. To investigate the network structure of the gene interactions, we used the Cytoscape3.71 tool to perform the visualization [40]. In addition, we used the Cytoscape plug-in tool CytoHubba to detect the core genes and survey potential critical driver genes in grapevine berry ripening.

## 3. Results

### 3.1. Quantitative Modeling for System Fluctuations of Berry Ripening

**Figure 1:**
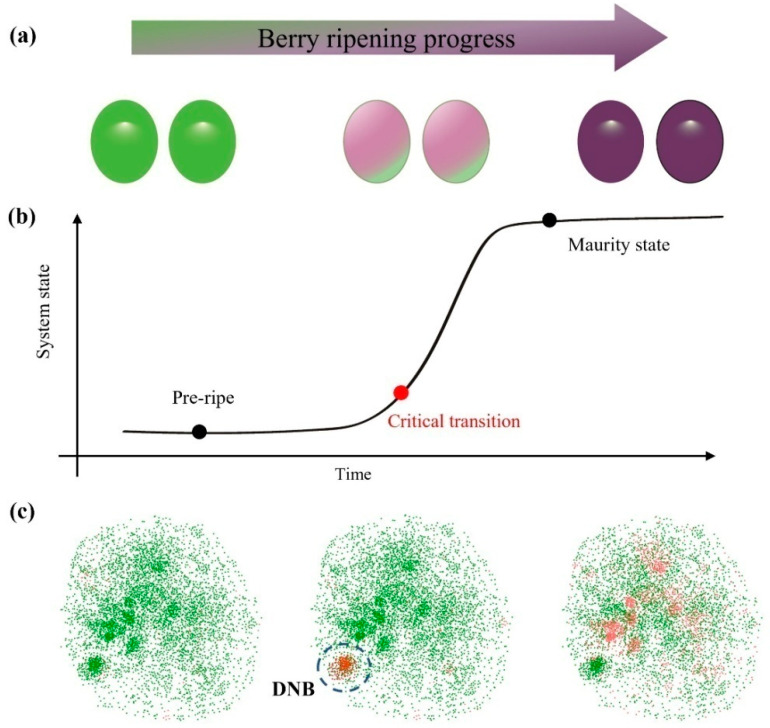
The diagram for the phase transition of berry/fruit ripening process. (**a**) The fruit development process and fruit ripening from pre-ripe to mature includes three phases. (**b**) The system in the pre-ripening phase and the maturity phase is smooth and steady. Meanwhile, in the critical transition state, the system fluctuates, and the transition driver genes fluctuate in their expression. (**c**) The peak of the PSI index for DNB module indicates the tipping point of the phase transition.

Grapevine fruit ripening is a complex biological process involving a phase transition from immature to mature in terms of physiological and morphological characteristics. The first stage is the pre-ripe state, which is characterized by berry formation, fruit enlargement and organic acid accumulation. The second stage is the critical transition of fruit ripening, which is characterized by berry veraison, discoloration and softening. The third stage is fruit after-ripening, which is characterized by increased sugar and aroma accumulation. As an important event in fruit development and ripening, the phase transition from before to after coloration is usually rapid and qualitatively changes the fruit ripening system. Previous studies have shown that physiology and gene expression of grape berries are completely different before and after veraison (Figure 1a).

During the fruit ripening process, the system in the pre-ripening phase and the maturity phase is smooth and steady, and the genes in the genome-wide analysis are normally expressed. Meanwhile, in the critical transition state, the system fluctuates, and the transition driver genes fluctuate in their expression (Figure 1b). To detect the critical transition event of fruit development, and to investigate the regulation of fruit ripening in grapevine, dynamic network analysis was performed using the dynamic network biomarker (DNB) analysis method. As a dynamical systems theory-based method, the DNB method can identify the critical transition point and a cluster of genes as the regulators of phase transition. To define this event quantitatively, time-course gene expression is used for the computation.

The DNB genes can be identified using the numerical signal of following phase transition scoring index (PSI), i.e.,

$$\mathrm{PSI}=\mathrm{SD}i*\frac{\mathrm{PCC}i}{\mathrm{PCC}o}$$

where PCC*i* is the weighted Pearson’s correlation coefficient of genes in the putative DNB module, PCC*o* is the weighted Pearson’s correlation coefficient of genes that are outside of the putative DNB module genes, and SD*i* is the weighted standard deviation of gene expression in the putative DNB module. The PSI index can capture the fluctuating trait of genes controlling the phase transition of systems. The peak of the PSI index indicates the tipping point of phase transition (Figure 1c). The difference between this and general methods is that the DNB method can measure the fluctuation change of gene modules based on genome-wide associations.

### 3.2. Dynamic Network Biomarker Analysis of Grapevine Fruit Ripening

**Figure 2:**
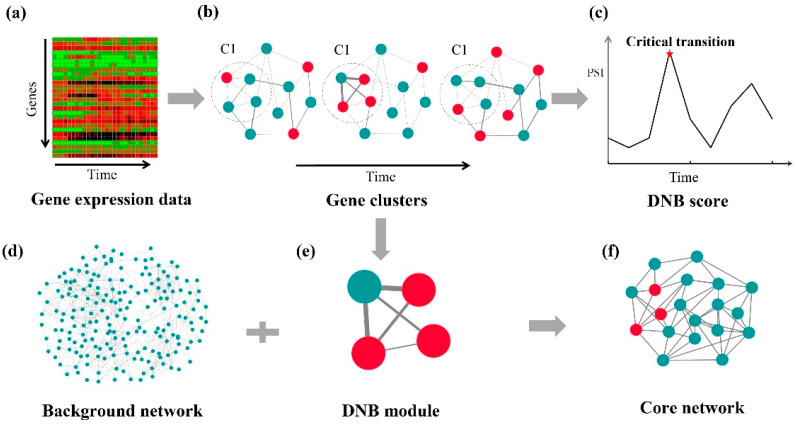
The flowchart of dynamic network analysis of grapevine fruit ripening.(**a**) The time-series gene expression of grapevine fruit ripening. (**b**) For each time point, the genes were clustered into modules using the clustering algorithm and then the gene modules were used as a candidate DNB module for the regulator of phase transition of grapevine fruit ripening. (**c**) With these candidate gene modules, the PSI index was computed for each gene module in each sample. The module with the highest PSI index was collected for each sample and then these maximum PSI indices were used to detect the critical transition. (**d**) The background network of the PPIs from STRING. (**e**) The gene module with the maximum PSI index was taken as the DNB module. (**f**) The core network for grapevine fruit ripening was constructed with background network.

To identify the critical transition of grapevine fruit ripening, the dynamic network biomarker (DNB) method was performed on time-series transcriptome data. As the outputs of the analysis, a tipping point and a DNB module were obtained. The flowchart for the analysis of grapevine fruit ripening was shown in Figure 2.

To obtain gene expression at different times during the fruit development, read alignment and assembly were implemented using RNA-Seq (Figure 2a). With gene expression values (Supplementary Table S1), the dynamic network analysis was performed to investigate grapevine fruit ripening. For each sample, the genes were clustered into modules by using the clustering algorithm, and then the gene modules were used as candidate DNB modules, i.e., the regulators of phase transition of grapevine berry ripening (Figure 2b). With these candidate gene modules, the PSI indices were computed for each gene module in each sample. The module with the highest PSI index was collected for each sample, and then these maximum PSI indices were used to detect the critical transition point (Figure 2c). With the maximum PSI index, the module was taken as the DNB module, i.e., dynamic network biomarker (Figure 2e). To construct the core gene network of grapevine fruit ripening, both the DNB module genes and the differentially expressed genes were integrated with the background network of the protein–protein interactions from STRING (Figure 2d–f).

### 3.3. Critical Transition of Grapevine Berry Ripening

**Figure 3:**
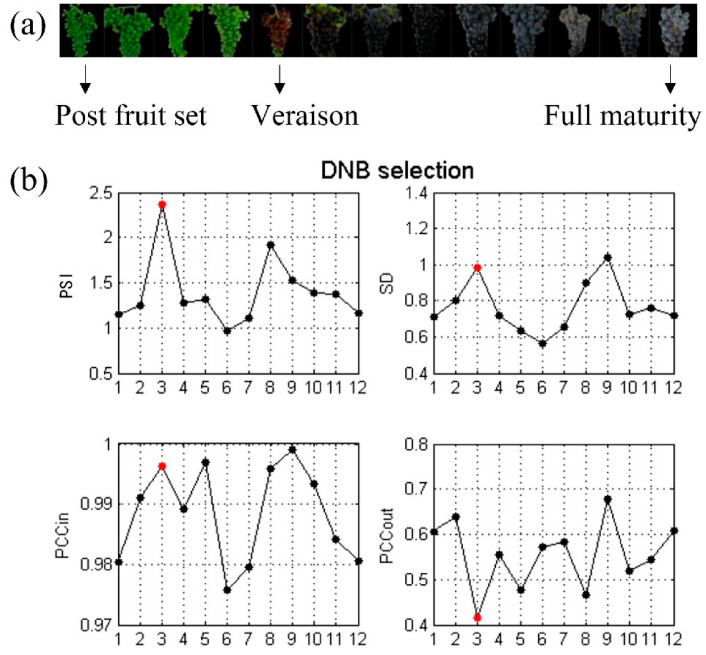
Grapevine berry ripening progress and comparison of gene modules with the highest comprehensive scores in each period. (**a**) The progress of grape berry ripening. (**b**) Dynamic changes inthe comprehensive index ofthe DNB module. The third time point with the maximum PSI index value was recognized as the critical transition point of grapevine berry ripening. The red point represents the tipping point of the systems.

The time-series RNA-seq data of Sauvignon grapes at 13 time points from post setting fruit to full ripening were analyzed to detect the critical transition of fruit ripening and to identify the associated genes (Figure 3a). To match the data requirement for data analysis using the DNB method, the former and the latter samples were used as case and control samples. With this principle, a total of 12 sets data were obtained. The grapevine berries grown in the same location were collected from the setting fruit to harvest at the same time of day in three consecutive years. The grape ripening process shows three distinct stages of a double growth curve in terms of physiological and morphological characteristics. At the transition of grape berry ripening, the phenotypes of berries were from slow-growing and coloring to the increase in sugar and the accumulation of aroma.

As shown in Figure 3b, the third time point (corresponding to the phenotypic data of the fourth time point) with the maximum PSI index value was recognized as the critical transition point of grapevine fruit ripening. Since veraison is defined as a change in color in half of a cluster of grapes, the identified key transition points coincided with the onset of the veraison of grape fruit ripening. The comparison of the PSI indices of the DNB module at different time points showed that the PSI index was highest at the critical transition point. The weighted SDs and PCCs of genes in the identified DNB module were high, while the weighted Pearson’s correlation coefficient outside of the DNB module genes was low.

### 3.4. Characteristics and Core Networks of the DNB Genes

Apart from the critical transition point of the results, a module with 68 genes was detected as a dynamic network biomarker for grapevine fruit ripening. To investigate the functions of DNB genes in the phase transition of grapevine fruit ripening, GO (Gene Ontology) enrichment analysis was performed.

**Figure 4:**
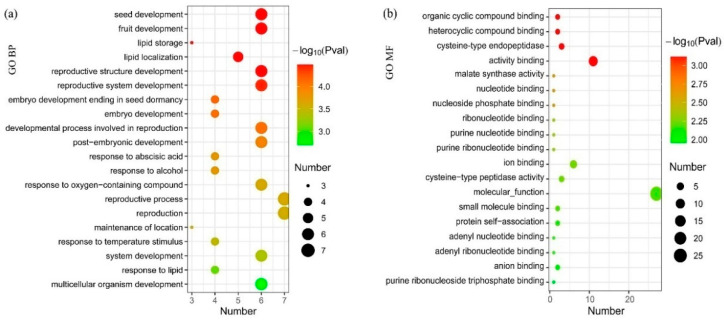
GO enrichment analysis of DNB genes. (**a**) In the results of the GO analysis, ‘seed development’, ‘fruit development’ and ‘lipid storage’ were the top GO terms. (**b**) ‘Organic cyclic compound binding’, ‘heterocyclic compound binding’, and ‘cysteine-type endopeptidase activity’ were significantly enriched in the molecular functional category.

In the results of GO analysis, ‘seed development’, ‘fruit development’ and ‘lipid storage’ were the top GO terms in the biological process category. Additionally, GO terms such as ‘lipid localization’ and ‘reproductive system development’ were also significantly enriched (Figure 4a). ‘Organic cyclic compound binding’, ‘heterocyclic compound binding’, and ‘cysteine-type endopeptidase activity’ were enriched in the molecular functional category significantly (Figure 4b).In addition, ‘malate synthase activity’ and ‘carbohydrate derivative binding’ were also enriched in the molecular functional category. Significantly enriched cellular components include ‘monolayer-surrounded lipid storage body’, ‘nucleus’, and ‘lysosomes’. Supplementary Table S2 provides all GO terms of the enrichment analysis of DNB genes, such as the cellular component, molecular function, and biological process.

**Figure 5:**
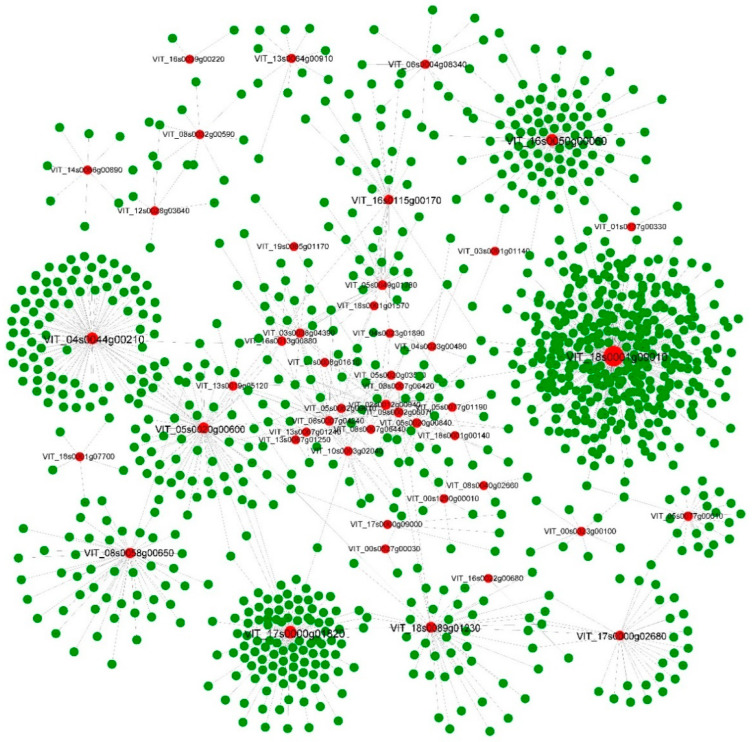
The core network of regulators includes the inner network of the DNB genes and the outer network ofDNB genes and their surrounding genes.

To show the core network of the DNB genes, we constructed the inner network of the DNB genes and the outer network between the DNB genes and their surrounding genes. As a result, 287 nodes and 708 edges were involved in the core network, including inner and outer networks of DNB genes (Figure 5).The DNB genes and the surrounding genes that interact with them are noted in red and green respectively. Further analysis showed that the gene with the highest connectivity, VIT_18s0001g09010, interacted with 358 genes. The gene with the second highest degree of connectivity, VIT_17s0000g01820, interacted with 107 genes, and the gene function annotation showed that the gene was malate synthase, and was involved in the metabolism of sugar and acid. In addition, it was found that genes such as VIT_09s0002g06070, VIT_16s0115g00170, and VIT_05s0020g00840 are involved in the seed maturation process in the highly connected genes, implying that seed ripening is related to the berry ripening process.

### 3.5. Differentially Expressed Genes between Maturity and Pre-Rip State

**Figure 6:**
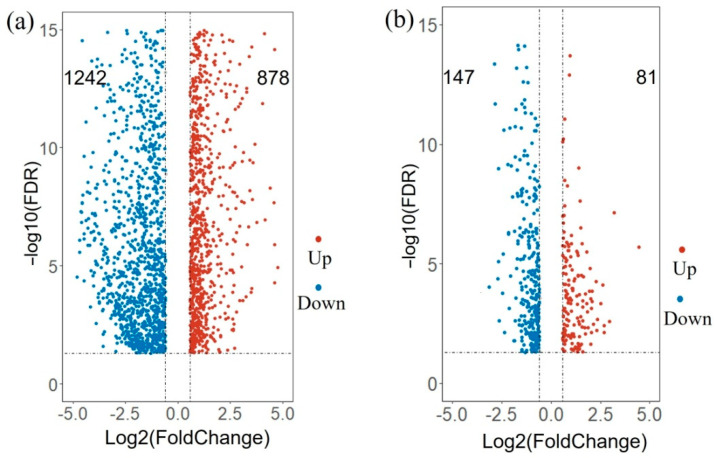
The Volcano plot displaying DEGs for the transition of grapevine berry ripening. (**a**) The differentially expressed genes between T4 and T2. (**b**) The differentially expressed genes between T9 and T7.

To study the function of the differentially expressed genes (DEGs) between the pre-rip and the maturity states(T4/T2), the genes with expression changes before and after the key transition were obtained by differential analysis. The results showed that about 2120 genes were differentially expressed during the transition period, 878 genes of which were up-regulated, and 1242 genes of which were down-regulated (Figure 6a).

We also analyzed the differential expression of genes before and after the eighth time point (T9/T7), where the second PSI peak was identified. The results showed that about 228 genes were differentially expressed, among which 81 and 147 were up- and down-regulated, respectively (Figure 6b). This is consistent with previous findings, with more genes differentially expressed at the first transition point for the onset of fruit verasion. The DEGs between the fourth time point and the second time point are listed in Supplementary Table S3, and the DEGs between the 9th and 7th time points are listed in Supplementary Table S4.

The GO enrichment analysis results showed that at the first transition, 873 of the 878 up-regulated genes were assigned GO terminology. Among them, “gene expression”, “nucleic acid metabolic process”, “carbohydrate derivative metabolic process” and “carbohydrate metabolic process” were significantly enriched in the biological process category. Terms including “RNA binding”, “nucleic acid binding”, “oxoreductase activity”, and “structural molecular activity” were significantly enriched in the molecular functional category. In the cell component category, “protein-containing complex”, “nuclear protein-containing complex” and “non-membrane-bounded organelle intracellular” were significantly enriched. All of the significantly enriched GO terms are listed in Supplementary Table S5. At the second transition point, 73 of the 81 up-regulated genes were assigned to the GO terms. Among them, biological process categories including “nitrogen compound metabolic process”, “cell metabolism process”, “macromolecular metabolism process”, “organic matter metabolism process” and other terms were significantly enriched. Terms such as “catechol oxidase activity”, “carbohydrate derivative synthesis”, and “selenium binding” were significantly enriched in the molecular functional category. In addition, terms such as “cellular component”, “cellular anatomical entity”, and “intracellular organelle” were significantly enriched in the cell component category. Supplementary Table S6 lists all of the significantly enriched GO terms, including the cellular component, molecular function, and biological process categories.

## 4. Discussion

Under evolution theory, to facilitate the dispersal of seeds in different ecological environments and times, fruits have evolved in various forms. The phase transition of fruit ripening from immature to mature represents a dramatic shift in survival strategies. This transition is very important for fleshy fruits, in order for them to be attractive to seed-scattering animals while protecting themselves from animals [41,42]. Therefore, to reach optimal fitness, maturity must be tightly controlled and coordinated with seed development [43,44]. The time and speed of ripening are two key factors that influence grapevine berry quality, and the ripening process and harvest maturity decide the flavor compounds in grapes [45]. The onset of veraison is considered to be a key determinant of berry harvest date, but little is known regarding initiating factors of the critical phase transition [46,47].

In this work, we used an integrated dynamic network biomarker analysis approach with non-model plant species in order to identify the critical transition points in fruit ripening based on time-series RNA-seq data of grapevine berry ripening. The results showed that the third time point was identified as the critical transition point for fruit ripening, corresponding to the shift from the first stage of the grapevine berry (acid accumulation and fruit enlargement) to the second stage of color change. In addition, the second peak of the dynamic network analysis composite index score also corresponded to the transition from the second to the third stage of berry development (softening of firmness, accumulation of aromatic substances). The analysis showed that grapes have completely different transcriptional and metabolic strategies at different stages. Unlike the steady state of each stage, the system of fruit development is usually not smooth or nonlinear during the transition stage. Plants accumulate acid in the first stage, the system is stable during fruit enlargement, and the system fluctuates violently at the critical transition point of fruit ripening (color transformation), including signal transmission, gene expression changes, metabolic pathway redistribution, and resource redistribution. The differential expression analysis results showed that at the two transition points, there were 2120 differentially expressed genes at the first transition point and 228 differentially expressed genes at the second transition point.

The results of the GO enrichment analysis of up-regulated expression genes of two transition points showed that at the first transition point, from the growth phase to the lagging stage, the genes involved in biological processes such as carbohydrate metabolism and macromolecular metabolism were up-regulated. This is consistent with existing research reporting that the process of primary carbohydrate metabolism, which includes sucrose synthesis and degradation, glycolysis, and the tricarboxylic acid (TCA) cycle, playsa central role in the ripening transition and final composition of the berries [48,49]. During the growth phase, berries accumulate organic acids, which remain green and hard, while sugar begins to accumulate during the late lag period, and the content of organic acids and tannins decreases. During the second transition point of the transition from lag to maturation, up-regulated expression genes are widely involved in the metabolism of nitrogen-containing compounds and cell metabolism. During maturation, the fruit synthesizes aromatic compounds, berries soften, and their attractiveness to animals is increased [7].

Based on dynamic network analysis, we also identified 68 genes that were considered key genes for critical transitions in fruit ripening. The GO (Gene Ontology) enrichment analysis showed that these genes were correlated with fruit and seed development. This result validated previous findings that, during grapevine berry development, the end of seed development coincides with the start of berry ripening [50,51].Compared with the seeded berries in Pinot Noir and Cabernet Sauvignon clusters, the seedless berries grew at a lower rate, and fruits with the same seed content started ripening at the same time [52]. According to known molecular mechanisms, auxin is synthesized and then transported to the pericarp, and induces biosynthesis of gibberell, which promotes ovarian growth and fruit development [53,54].However, as in parthenocarpic and seedless fruits, fruit development can be decoupled from seed development. This suggests that phytohormones may come from sources other than seeds, or that, while seed and fruit development are regulated by the same mechanism, the two processes can be uncoupled [50]. There are some factors controlling fruit ripening, such as the external environment, a variety of hormones, and a complex regulatory network of many transcription factors. In future, refined molecular mechanisms need to be studied with the use of datasets with higher temporal and spatial resolution [55,56].

## 5. Conclusions

In conclusion, different from the previous physiological and biochemical studies of various stages of fruit ripening, this study provides a new perspective for understanding the critical transitions between the various stages of grape berry ripening. This study shows that grapevine berry ripening is closely correlated with seed development. As a result, we provide some candidate genes for marking fruit ripening. On the basis of the functional analysis, these genes are involved in the molecular event of grapevine berry ripening. We also provide references for other research on the transition of fruit ripening in climacteric and non-climacteric fruits. With the development of single-cell sequencing technology and reductions in sequencing costs, higher-resolution spatiotemporal transcriptome data from different tissues will also provide great assistance in understanding the molecular mechanism of the phase transition of fruit ripening.

## Data Availability

The data supporting the findings of this study are included in the supplementary files.

## Supplementary Materials

The following supporting information can be downloaded at: https://www.mdpi.com/article/10.3390/genes13101851/s1, Table S1: The expression of DNB genes; Table S2: The GO enrichment of DNB genes; Table S3: The differential expressed genes between T4 and T2; Table S4: The differential expressed genes between T9 and T7; Table S5: GO enrichment analysis results of up-regulated expressed genes at grape berry ripening first transition; Table S6: GO enrichment analysis results of up-regulated expressed genes at grape berry ripening second transition.

## Abbreviations

ABA	Abscisic acid
BR	Brassinosteroid
DNB	Dynamic network biomarker
DEG	Differentially expressed gene
FC	Fold change
FDR	False discovery rate
RPKM	Reads Per Kilobase per Million mapped reads
GO	Gene ontology
PCC	Pearson correlation coefficient
PPI	Protein–protein interaction
PSI	Phase transition scoring index
SD	Standard deviation
WGCNA	Weighted correlation network analysis

## References

[1] Coombe B.G., Hale C.R. (1973). The hormone content of ripening grape berries and the effects of growth substance treatments. Plant Physiol..

[2] Foster J.D. (2018). Grape, raisin, and lily ingestion. Textbook of Small Animal Emergency Medicine.

[3] Berhe D.T., Belew D. (2022). Evaluation of wild, wine, table, and raisin grapevine (Vitis spp.) genotypes in Gedeo Zone, Southern Ethiopia. Sci. World J..

[4] Kuhn N., Guan L., Dai Z.W., Wu B.-H., Lauvergeat V., Gomès E., Li S.-H., Godoy F., Arce-Johnson P., Delrot S. (2013). Berry ripening: Recently heard through the grapevine. J. Exp. Bot..

[5] Coombe B.G. (1992). Research on development and ripening of the grape berry. Am. J. Enol. Vitic..

[6] Zhang A., Zhou H., Jiang X., Han Y., Zhang X. (2021). The draft genome of a flat peach (Prunus persica L. cv.‘124 Pan’) provides insights into its good fruit flavor traits. Plants.

[7] Deluc L.G., Grimplet J., Wheatley M.D., Tillett R.L., Quilici D.R., Osborne C., Schooley D.A., Schlauch K.A., Cushman J.C., Cramer G.R. (2007). Transcriptomic and metabolite analyses of Cabernet Sauvignon grape berry development. BMC Genom..

[8] COOMBE B.G., McCARTHY M. (2000). Dynamics of grape berry growth and physiology of ripening. Aust. J. Grape Wine Res..

[9] Fasoli M., Richter C.L., Zenoni S., Bertini E., Vitulo N., Dal Santo S., Dokoozlian N., Pezzotti M., Tornielli G.B. (2018). Timing and order of the molecular events marking the onset of berry ripening in grapevine. Plant Physiol..

[10] D’Incà E., Cazzaniga S., Foresti C., Vitulo N., Bertini E., Galli M., Gallavotti A., Pezzotti M., Tornielli G.B., Zenoni S. (2021). VviNAC33 promotes organ de-greening and represses vegetative growth during the vegetative-to-mature phase transition in grapevine. New Phytol..

[11] Zhang A., Xiong Y., Fang J., Jiang X., Wang T., Liu K., Peng H., Zhang X. (2022). Diversity and Functional Evolution of Terpene Synthases in Rosaceae. Plants.

[12] Cherian S., Figueroa C.R., Nair H. (2014). ‘Movers and shakers’ in the regulation of fruit ripening: A cross-dissection of climacteric versus non-climacteric fruit. J. Exp. Bot..

[13] Chen Y., Grimplet J., David K., Castellarin S.D., Terol J., Wong D.C.J., Luo Z., Schaffer R., Celton J.M., Talon M. (2018). Ethylene receptors and related proteins in climacteric and non-climacteric fruits. Plant Sci..

[14] Jia H.F., Lu D., Sun J.H., Li C.L., Xing Y., Qin L., Shen Y.Y. (2013). Type 2C protein phosphatase ABI1 is a negative regulator of strawberry fruit ripening. J. Exp. Bot..

[15] Pilati S., Bagagli G., Sonego P., Moretto M., Brazzale D., Castorina G., Simoni L., Tonelli C., Guella G., Engelen K. (2017). Abscisic acid is a major regulator of grape berry ripening onset: New insights into ABA signaling network. Front. Plant Sci..

[16] Symons G.M., Davies C., Shavrukov Y., Dry I.B., Reid J.B., Thomas M.R. (2006). Grapes on steroids. Brassinosteroids are involved in grape berry ripening. Plant Physiol..

[17] Böttcher C., Burbidge C.A., Boss P.K., Davies C. (2013). Interactions between ethylene and auxin are crucial to the control of grape (*Vitis vinifera* L.) berry ripening. BMC Plant Biol..

[18] Kapoor L., Simkin A.J., George Priya Doss C., Siva R. (2022). Fruit ripening: Dynamics and integrated analysis of carotenoids and anthocyanins. BMC Plant Biol..

[19] Chen T., Qin G., Tian S. (2020). Regulatory network of fruit ripening: Current understanding and future challenges. New Phytol..

[20] García-Gómez M.L., Castillo-Jiménez A., Martínez-García J.C., Álvarez-Buylla E.R. (2020). Multi-level gene regulatory network models to understand complex mechanisms underlying plant development. Curr. Opin. Plant Biol..

[21] Deng Z., Zhang J., Li J., Zhang X. (2021). Application of Deep Learning in Plant–Microbiota Association Analysis. Frontiers in Genetics.

[22] Kuang J.F., Wu C.J., Guo Y.F., Walther D., Shan W., Chen J.Y., Chen L., Lu W.J. (2021). Deciphering transcriptional regulators of banana fruit ripening by regulatory network analysis. Plant Biotechnol. J..

[23] Shinozaki Y., Nicolas P., Fernandez-Pozo N., Ma Q., Evanich D.J., Shi Y., Xu Y., Zheng Y., Snyder S.I., Martin L.B. (2018). High-resolution spatiotemporal transcriptome mapping of tomato fruit development and ripening. Nat. Commun..

[24] Zhang X., Zhao X.-M., He K., Lu L., Cao Y., Liu J., Hao J.-K., Liu Z.-P., Chen L. (2012). Inferring gene regulatory networks from gene expression data by path consistency algorithm based on conditional mutual information. Bioinformatics.

[25] Zhang X.J., Liu K.Q., Liu Z.P., Duval B., Richer J.M., Zhao X.M., Hao J.K., Chen L.N. (2013). NARROMI: A noise and redundancy reduction technique improves accuracy of gene regulatory network inference. Bioinformatics.

[26] Zhang X., Zhao J., Hao J.-K., Zhao X.-M., Chen L. (2015). Conditional mutual inclusive information enables accurate quantification of associations in gene regulatory networks. Nucleic Acids Res..

[27] Zhao J., Zhou Y., Zhang X., Chen L. (2016). Part mutual information for quantifying direct associations in networks. Proc. Natl. Acad. Sci..

[28] Jiang X., Zhang X. (2022). RSNET: Inferring gene regulatory networks by a redundancy silencing and network enhancement technique. BMC Bioinform..

[29] Langfelder P., Horvath S. (2008). WGCNA: An R package for weighted correlation network analysis. BMC Bioinform..

[30] Li M., Zeng T., Liu R., Chen L. (2013). Detecting tissue-specific early warning signals for complex diseases based on dynamical network biomarkers: Study of type 2 diabetes by cross-tissue analysis. Brief. Bioinform..

[31] Liu R., Wang J., Ukai M., Sewon K., Chen P., Suzuki Y., Wang H., Aihara K., Okada-Hatakeyama M., Chen L. (2019). Hunt for the tipping point during endocrine resistance process in breast cancer by dynamic network biomarkers. J. Mol. Cell Biol..

[32] Wang T., Zhang X. (2021). Genome-wide dynamic network analysis reveals the potential genes for MeJA-induced growth-to-defense transition. BMC Plant Biol..

[33] Zhang F., Liu X., Zhang A., Jiang Z., Chen L., Zhang X. (2019). Genome-wide dynamic network analysis reveals a critical transition state of flower development in Arabidopsis. BMC Plant Biol..

[34] He S., Wang H., Hao X., Wu Y., Bian X., Yin M., Zhang Y., Fan W., Dai H., Yuan L. (2021). Dynamic network biomarker analysis discovers IbNAC083 in the initiation and regulation of sweet potato root tuberization. Plant J..

[35] Yang B., Li M., Tang W., Liu W., Zhang S., Chen L., Xia J. (2018). Dynamic network biomarker indicates pulmonary metastasis at the tipping point of hepatocellular carcinoma. Nat. Commun..

[36] Pertea M., Kim D., Pertea G.M., Leek J.T., Salzberg S.L. (2016). Transcript-level expression analysis of RNA-seq experiments with HISAT, StringTie and Ballgown. Nat. Protoc..

[37] Leng N., Dawson J.A., Thomson J.A., Ruotti V., Rissman A.I., Smits BM G., Haag J.D., Gould M.N., Stewart R.M., Kendziorski C. (2013). EBSeq: An empirical Bayes hierarchical model for inference in RNA-seq experiments. Bioinformatics.

[38] Mi H., Muruganujan A., Ebert D., Huang X., Thomas P.D. (2019). PANTHER version 14: More genomes, a new PANTHER GO-slim and improvements in enrichment analysis tools. Nucleic Acids Res..

[39] Szklarczyk D., Gable A.L., Lyon D., Junge A., Wyder S., Huerta-Cepas J., Simonovic M., Doncheva N.T., Morris J.H., Bork P. (2019). STRING v11: Protein-protein association networks with increased coverage, supporting functional discovery in genome-wide experimental datasets. Nucleic Acids Res..

[40] Shannon P., Markiel A., Ozier O., Baliga N.S., Wang J.T., Ramage D., Amin N., Schwikowski B., Ideker T. (2003). Cytoscape: A software environment for integrated models of biomolecular interaction networks. Genome Res..

[41] Bolmgren K., Eriksson O. (2010). Seed mass and the evolution of fleshy fruits in angiosperms. Oikos.

[42] Eriksson O. (2016). Evolution of angiosperm seed disperser mutualisms: The timing of origins and their consequences for coevolutionary interactions between angiosperms and frugivores. Biol. Rev..

[43] Brumos J. (2021). Gene regulation in climacteric fruit ripening. Curr. Opin. Plant Biol..

[44] Forlani S., Mizzotti C., Masiero S. (2021). The NAC side of the fruit: Tuning of fruit development and maturation. BMC Plant Biol..

[45] He L., Ren Z.Y., Wang Y., Fu Y.Q., Li Y., Meng N., Pan Q.H. (2020). Variation of growth-to-ripening time interval induced by abscisic acid and synthetic auxin affecting transcriptome and flavor compounds in Cabernet Sauvignon grape berry. Plants.

[46] Töpfer R., Hausmann L., Harst M., Maul E., Zyprian E., Eibach R. (2011). New horizons for grapevine breeding. Fruit Veg. Cereal Sci. Biotechnol..

[47] Falchi R., Wong D.C., Yan Y., Savoi S., Gambetta G.A., Castellarin S.D. (2019). The genomics of grape berry ripening. The Grape Genome.

[48] Dai Z.W., Léon C., Feil R., Lunn J.E., Delrot S., Gomès E. (2013). Metabolic profiling reveals coordinated switches in primary carbohydrate metabolism in grape berry (*Vitis vinifera* L.), a non-climacteric fleshy fruit. J. Exp. Bot..

[49] Centeno D.C., Osorio S., Nunes-Nesi A., Bertolo A.L., Carneiro R.T., Araújo W.L., Steinhauser M.C., Michalska J., Rohrmann J., Geigenberger P. (2011). Malate plays a crucial role in starch metabolism, ripening, and soluble solid content of tomato fruit and affects postharvest softening. Plant Cell.

[50] Gouthu S., Deluc L.G. (2015). Timing of ripening initiation in grape berries and its relationship to seed content and pericarp auxin levels. BMC Plant Biol..

[51] Ristic R., Iland P.G. (2005). Relationships between seed and berry development of Vitis Vinifera L. cv Shiraz: Developmental changes in seed morphology and phenolic composition. Aust. J. Grape Wine Res..

[52] Vondras A.M., Gouthu S., Schmidt J.A., Petersen A.R., Deluc L.G. (2016). The contribution of flowering time and seed content to uneven ripening initiation among fruits within *Vitis vinifera* L. cv. Pinot noir clusters. Planta.

[53] He H., Yamamuro C. (2022). Interplays between auxin and GA signaling coordinate early fruit development. Horticulture Research.

[54] Cosme F., Gonçalves B., Inês A., Jordão A.M., Vilela A. (2016). Grape and wine metabolites: Biotechnological approaches to improve wine quality. Grape Wine Biotechnol..

[55] Li Z., Jiang G., Liu X., Ding X., Zhang D., Wang X., Zhou Y., Yan H., Li T., Wu K. (2020). Histone demethylase SlJMJ6 promotes fruit ripening by removing H3K27 methylation of ripening-related genes in tomato. New Phytol..

[56] Ding X., Liu X., Jiang G., Li Z., Song Y., Zhang D., Jiang Y., Duan X. (2021). SlJMJ7 orchestrates tomato fruit ripening via crosstalk between H3K4me3 and DML2-mediated DNA demethylation. New Phytol..

